# A review of the evidence for the canonical Wnt pathway in autism spectrum disorders

**DOI:** 10.1186/2040-2392-3-10

**Published:** 2012-10-19

**Authors:** Hans Otto Kalkman

**Affiliations:** 1 Neuroscience Department, Novartis Institute of Biomedical Research, Building 386-14.22.15, Basel, CH 4002, Switzerland

**Keywords:** WNT2, FZD9, BCL9, DOCK4, DISC1, ADAM10, Valproate, SSRI

## Abstract

Microdeletion and microduplication copy number variations are found in patients with autism spectrum disorder and in a number of cases they include genes that are involved in the canonical Wnt signaling pathway (for example, FZD9, BCL9 or CDH8). Association studies investigating WNT2, DISC1, MET, DOCK4 or AHI1 also provide evidence that the canonical Wnt pathway might be affected in autism. Prenatal medication with sodium-valproate or antidepressant drugs increases autism risk. In animal studies, it has been found that these medications promote Wnt signaling, including among others an increase in Wnt2 gene expression. Notably, the available genetic information indicates that not only canonical Wnt pathway activation, but also inhibition seems to increase autism risk. The canonical Wnt pathway plays a role in dendrite growth and suboptimal activity negatively affects the dendritic arbor. In principle, this provides a logical explanation as to why both hypo- and hyperactivity may generate a similar set of behavioral and cognitive symptoms. However, without a validated biomarker to stratify for deviant canonical Wnt pathway activity, it is probably too dangerous to treat patients with compounds that modify pathway activity.

## Introduction

Autism is a developmental disorder that appears in the first three years of life. Diagnostic behavioral symptoms of autism are abnormal socialization, limited communication, unusually narrow interests and repetitive behaviors [1,2]. Clinical presentation and intellectual abilities are, however, extremely heterogeneous and autism may be better described as autism spectrum disorders. There is a large difference in concordance rates between monozygotic and dizygotic twins, which indicates that autism spectrum disorders have a strong genetic basis [3-5]. Investigations of genome-wide single nucleotide polymorphisms and copy number variations have generated a long list of candidate genes [6-8]. These candidate genes have very diverse functions and interactions [9-12] and the process by which these modified genes contribute or cause autism remains poorly understood. One approach to shed some light on pathological processes is to arrange the identified candidate genes according to place and function within known intracellular signal transduction cascades. When the functional consequence of a given mutation is known, one can infer whether the signaling pathway is activated or suppressed, and ultimately one can try to estimate the functional consequences in terms of neuronal function, brain circuits and behavioral output. In the present report, mutations in the canonical Wnt (wingless-type MMTV integration site) pathway that are found in cases with autism are reviewed. Before going into detail, it is important to sketch this Wnt signal transduction cascade.

## Description of the canonical Wnt pathway

**Figure 1:**
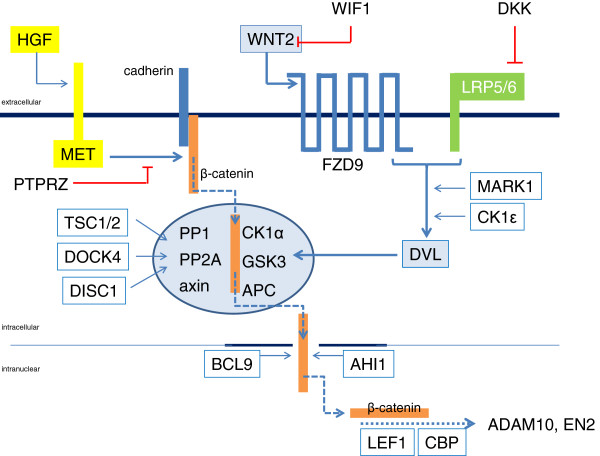
**Schematic representation of the canonical Wnt2 pathway, including all genes discussed in the current review.** Wnt2 activates the 7-transmembrane-spanning Fzd9 receptor, which together with the co-receptor LRP5/6 activates dishevelled (DVL). Activated DVL inhibits the activity of the β-catenin “destruction complex” (indicated as a light blue ellipse). β-catenin is released from its complex with cadherin by the activity of the HGF receptor MET. When β-catenin is protected against destruction, it can enter the nucleus, bind the transcription factor LEF1 and co-factors to promote transcription of target genes like, for example, engrailed 2 (*EN2*). The functional consequence is an increase in cell growth and motility.

For a rapid overview of the canonical Wnt pathway, the reader may consult the cartoon in Figure 1. ‘Wnts’ are lipid-modified signaling proteins that act as short range ligands to activate receptor-mediated signaling cascades. In mammals, some 19 Wnt members exist [13]. The proteins which act as cell surface receptors for Wnts are called ‘frizzled’ and, of these, 10 members have been described. Activated frizzled receptors connect to several downstream pathways [14-16]. In the so-called canonical Wnt pathway, signal transduction involves a low-density lipoprotein co-receptor (either LRP5 or LRP6) and polymerization of a protein called ‘dishevelled’ (abbreviated DVL; three isoforms) [14,17-19]. Activation of the canonical Wnt pathway leads to dissociation of cadherin/β-catenin complexes in the cell membrane with release of β-catenin, a process involving the phosphorylation of the Tyr-142 residue of β-catenin by the hepatocyte growth factor receptor Met [20,21].

Once released from the membrane, the fate of β-catenin in the cytoplasm is determined by a multi-protein complex (frequently referred to as the ‘destruction complex’), consisting of two serine-threonine kinases (CK1α and GSK3), two scaffolding proteins (axin and APC) and the phosphatases PP1 and PP2A [15,16]. Depending on the strength of the Wnt signal, this complex either promotes the catabolism of axin or that of β-catenin [17].

The factors which determine the shuttling of β-catenin between cytoplasm and nucleus are not entirely clear and its distribution seems to be determined by both cytosolic-retention factors (for example, axin, APC, cadherin) and nuclear retention factors (for example, BCL9) [20,22,23]. Within the nucleus, β-catenin again participates in several complexes, in this case consisting of high-mobility group (HMG) transcription factors (TCF7L 1–3; the latter is also called lymphoid enhancer factor-1; LEF1) and co-activators like for instance CREB binding protein (CREBBP) [24], PYGO [25] and BCL9 [25].

In mammalian species two isoforms of GSK3 exist (α, β). Of these, the β-isoform has a higher expression level and has been studied preferentially. Activity and cellular localization of GSK3 are regulated by phosphorylation steps. Autophosphorylation at Tyr-216 of GSK3β is required for full enzymatic activity [26]. Importantly, the protein encoded by the gene “disrupted in schizophrenia-1” (*DISC1*) directly interacts with GSK3β and suppresses Tyr-216-autophosphorylation [27] and contributes to effective canonical Wnt signaling.

Several negative regulators of the Wnt pathway are known as well. These negative regulators act by intercepting the extracellular Wnt, by blocking the frizzled receptor or by blocking the LRP co-receptor [28]. Intracellularly, pathway activity can be reduced by phosphatases like RPTP β/ζ [29], PP1 or PP2A [15]. The full complexity of the Wnt pathway is still evolving [19].

The canonical Wnt pathway plays an important role in brain development [30-35] and synaptic function [36-38]. It is, therefore, evident that mutations in Wnt pathway genes were suspected to contribute to autism spectrum disorders and to psychiatric disorders in general [39,40].

## Overview of mutations in Wnt pathway in patients with ASD (part 1)

Multiple genome-wide screens have found evidence for linkage to autism on several chromosomes [7,41,42]. These loci are frequently quite large, and contain numerous potential candidate genes. A typical example is the broad linkage peak on 7q31 area that maps over 200 genes [7,41,43]. Among the >200 genes there are several that play a role in Wnt signal transduction, for example, the gene encoding Wnt2, the hepatocyte growth factor receptor Met (which can contribute to Wnt signaling by phosphorylating β-catenin at position Tyr-142), the phosphatase PTPRZ1 (RPTP β/ζ which reverses Tyr-142 phosphorylation), the Wnt-target gene, engrailed2 (*EN2*) and a gene called *DOCK4* (a member of the extended β-catenin destruction complex). In the sections immediately below, the canonical Wnt2 cascade will be reviewed in detail, whereas additional genes involved in Wnt signaling will be discussed in Part 2 further down. The reader will note that the strength of the evidence for the individual genes varies considerably (actually, the evidence for individual genes is in no single case ‘compelling’; see Table 1). The main purpose of the present review is, however, not to assess the validity of the individual finding, but more globally, to overview the overall pathway activity and to evaluate and estimate whether signaling is decreased or enhanced.

**Table 1:** Canonical Wnt pathway genes mutated in autism – summary of the evidence

**Gene**	**Gene location**	**Discovery strategy**	**Replications**	**Preclinical support**
*APC*	5q21-q22	association study 75 unrelated patients	single case of APC deletion	APC’s functional role
*DISC1*	1q42	association study in 144 families	no	DISC1 function
*EN2*	7q36	association study in 3 datasets of 518 families	yes, but opposite haplotype	Wnt target gene
*MET*	7q31	association studies in 4 cohorts; microdeletion in 2 pts (involving >25 genes)	yes	post mortem expression; animal data
*WIF1*	12q14.3	GWAS in 26 extended families; linkage peak of ≥19 genes	no	
*MARK1*	14q41	GWAS in 116 families; SNPs in MARK1	no	transcription of MARK1 altered by SNPs
*CDH10*	5p14.1	GWAS in 780 families; SNPs between CDH9 and CDH10 highly significant	replication cohort by the same authors	
*WNT2*	7q31.2	GWAS study in 75 families; 2 families with missense mutation in Wnt2	one positive, also two negative studies	role of Wnt2 in midbrain development
*PTPRZ1*	7q31.3	single case with deletion CNV of 20 genes	no	
*CDH15*	16q24.3	genome scanning; deletion CNV of 3 genes	no	
*CDH13*	16q23.3	GWAS; deletion CNV of single gene	no	
*CDH8*	16q21	GWAS; detection of rare deletion CNV	no	data from KO mice
*DOCK4*	7q31.1	GWAS; microdeletion CNV	no, but dyslexia cases	biochemical data
*BCL9*	1q21	deletion and duplication CNVs (14 genes)	multiple	
*FZD9*	7q11.23	recognized syndrome; deletion and duplication CNVs (>20 genes)	yes, multiple	Wnt2 receptor
*AHI1*	6q23.3	recognized syndrome: mutation screening identified multiple disruptive mutations	yes, multiple	
*CREBBP*	16p13.3	recognized syndrome; microdeletion CNVs and disruptive mutations	multiple, also cases with microduplications	data from KO mice
*TSC1/2*	9q34 / 16p13.3	recognized syndrome: mutation screening identified numerous missense mutations	yes, multiple	data from KO mice

The genes have been ranked according to their discovery process. Association studies of candidate gene studies have frequently yielded false positive results and are considered relatively weak evidence. Genome wide association studies followed by specific investigation of genes in the ‘hot spot’ may be more reliable, but replications are crucial. Copy number variations may provide good evidence but the duplicated or deleted regions are generally large and usually contain several candidate genes. Copy number variations that involve only a few or even a single gene give a strong indication for a pathological role of those genes, but thus far such CNVs have been detected only very rarely and lack replication. The best evidence comes from CNVs that give rise to recognizable syndromes. Unfortunately, in this case the involved genes are not specific for the canonical Wnt pathway and modify other pathways as well. In some cases, there is circumstantial support for a given gene from biochemical- or whole animal studies. Abbreviations: CNV, copy number variation; GWAS, genome wide association study; SNP, single nucleotide polymorphism. For details, please refer to the individual section in the text.

### *WNT2* (7q31.2)

Given the localization within the autism ‘hotspot’ 7q31, the *WNT2* gene has been screened for non-synonymous mutations in autistic probands ascertained through the Collaborative Linkage Study of Autism by Wassink *et al.*[44]. The authors identified several variants that segregated with autism and severe language abnormality. Two subsequent linkage studies were not able to confirm the original findings [45,46]; however, a more recent extended study again found an association [47]. This study involved a case–control study of 9 **single-nucleotide polymorphisms** (SNPs) within the *WNT2* gene in 170 autism patients and 214 controls from Japan, and a follow-up of the positive results in a transmission disequilibrium test (TDT) in 98 Japanese autistic family trios. The significant associations from the initial part were replicated in the TDT part and the authors concluded that “*WNT2* is a strong candidate gene for autism” [47]. The function of Wnt2 has also been investigated in laboratory experiments. In ventral midbrain cultures, administration of Wnt2 protein increased proliferation of progenitors and the number of dopamine neurons, whereas the opposite was found in WNT2 knock-out mice [48]. Thus, a too-strong Wnt2 signaling could lead to enhanced midbrain dopamine function, which eventually might relate to the repetitive behaviors seen in autism patients.

### *FZD9* (7q11.23)

Immuno-precipitation experiments demonstrated that Wnt2 interacts with frizzled 3 (Fzd3) and *Fzd9*, while an antibody for Fzd9, but not an Fzd3-antibody precipitated Wnt2 [49]. This result indicates that *Fzd9* is the preferred receptor for Wnt2. Evidence that Wnt2 not only binds, but also activates *Fzd9* was provided by Karasawa *et al.*[50], who demonstrated that Wnt2 application to *Fzd9* expressing HEK293 cells led to phosphorylation of dishevelled-1 (DVL1) and β-catenin-mediated gene transcription.

The 7q11.23 area is known to be relevant for the Williams-Beuren syndrome (WBS) [51,52]. Copy number variants of the WBS-region are responsible for a complex neurological, cognitive and behavioral syndrome with frequent involvement of multiple additional other organ systems [53]. There are interesting similarities and differences in clinical features of patients with a 7q11.23 deletion compared to those with duplications (reviewed by [51]). Developmental delay, mental retardation and Attention Deficit Hyperactivity Disorder (ADHD) are found in both groups, but whereas patients with a deletion are excessively social and verbally skilled, patients carrying a duplication display severe delays in language development and deficits in social interaction [51,54,55]. Male duplication patients, furthermore, show hyperactivity, self-injury and aggression [54]. The critical region is approximately 1.4 to 1.5 Mb [52,53] and contains some 20 genes, including *FZD9*. The consequence of a loss of the Fzd9 receptor has been investigated in mice. Homozygous deletion of *FZD9* resulted in severe deficits in visuospatial learning and memory, in apoptosis in the dentate gyrus and in a lowered seizure threshold [56]. To a lesser extent, these effects were also observed in heterozygous FZD9-KO mice (that is, the situation analogous to WBS ‘deletion’ patients) [56]. There is evidence that Fzd9 is the main Fzd-subtype expressed on neuronal progenitor cells [57]. Given this information, it is conceivable that alterations in *FZD9* gene-dose contribute the behavioral phenotype of patients with 7q11.23 copy number variations.

### *BCL9* (1q21)

*BCL9* contributes to transduction of the Wnt signal by promoting transcriptional activity and nuclear retention of β-catenin [23,25,58]. *BCL9* is located on 1q21.1, an area for which, as for *FZD9*, both microduplications and microdeletions are described [59,60]. Mefford and colleagues [59] detected a duplication of 1q21.1 in 9 out of 5,218 patients with unexplained mental retardation, autism or congenital abnormalities. From these, 50% had autism or autistic behaviors; 62% had macrocephaly and mild dysmorphic features and in seven out of eight cases there was a delay in learning or development. Also, Brunetti-Pierri *et al.*[60] described cases with both duplications and deletions and confirm the presence of autism, dysmorphic features and seizures in each patient group. Aggression and ADHD were seen in both groups, while patients with a 1q21.1 microdeletion had notable short statures and microcephaly. The critical minimal area was determined to be about 1.35 Mb and contains 14 genes [60]. It is of note that both research groups consider hydrocephalus-inducing homologue 2 (*HYDIN2*) as the most likely candidate gene and do not discuss *BCL9*.

## Discussion (Part 1)

The data reviewed above in principle describe the canonical Wnt cascade for Wnt2: Wnt2 activates its preferred receptor (Fzd9) and the intracellular signaling ultimately leads to BCL9-assisted β-catenin-mediated gene-transcription (see Figure 1). Several aspects are noteworthy. Under the assumption that duplication-CNVs increase and deletions decrease Wnt pathway activity, it seems that both activation and inhibition is associated with autism. This observation is suggestive for a bell-shaped dose–response relationship between Wnt2 pathway activity and cognitive/linguistic development. But there are also differences between the groups. Patients with deletions of the Fzd9 genomic area are described to be highly socially active and empathic. This is in evident contrast with the aggression and social inhibition encountered in patients in whom the Wnt2 pathway (presumably) is overactive (Fzd9 duplication, *BCL9* duplication). The canonical Wnt pathway has an important influence on organ sizes, including the brain [35]. In agreement with this, microcephaly was noted in *BCL9* deletion patients, while macrocephaly was reported *BCL9* duplication patients. These results indicate a linear dose-dependency between Wnt pathway activity and skull size. In the following sections, further genes that influence canonical Wnt signaling will be reviewed.

## Overview of mutations in Wnt pathway in patients with ASD (Part 2)

### *WIF1* (12q14.3)

A genome-wide linkage analysis found autism to be linked to the 12q14 region [61]. The most significant linkage peak reason encompasses approximately 19 genes, including the Wnt inhibitory factor (*WIF1*). *WIF1* is mainly known for its function as tumor suppressor and disruptive mutations and/or epigenetic silencing enhanced cancer risk by activating Wnt signaling. It is, however, unknown if mutations in *WIF1* are responsible for the linkage to 12q14.

### *MARK1* (14q41)

*MARK1* (microtubule affinity regulating kinase-1, also: *PAR1*) is one of the kinases which phosphorylates and, thereby, activates dishevelled [62,63]. Several SNPs in the *MARK1* gene were associated with ASD and one of these SNPs was reported to affect transcription rate [64]. Consistent with this, increased mRNA levels of *MARK1* have been found in post mortem frontal cortex samples from autism subjects [64]. These data suggest that the autism-related mutations in *MARK1* activate the Wnt pathway. Interestingly, both overexpression and silencing of *MARK1* was found to result in shortened dendrites in mouse neocortical neurons, indicating a bell-shaped dose–response curve [64].

### *PTPRZ1* (7q31.3)

As described above, there are some 200 genes underneath the broad linkage peak on the long arm of chromosome 7, but fortunately specific copy number variations may allow some locus refinement. Quite recently a submicroscopic deletion of 5.4 Mb size was detected in a three-year-old boy with autism spectrum disorder that encompassed just 20 genes [65]. These 20 genes included the autism-candidate genes, *CADPS2* and *TSPAN12*, but also the receptor tyrosine phosphatase, RPTP β/ζ (*PTPRZ1*). Since *PTPRZ1* is a negative regulator of the Wnt pathway, the expected functional consequence of a haplo-insufficiency would be an increase in Wnt pathway activity. The clinical case description does, however, not fully support Wnt pathway hyperactivity since the patient’s head circumference was 2.4 SD below control and not, as expected for Wnt-pathway activation, above average.

### *MET* (7q31)

*MET* is the receptor for hepatocyte growth factor (HGF). Activated by HGF, *MET* phosphorylates membrane-bound β-catenin at Tyr-142, which promotes dissociation of the β-catenin/cadherin complex [21,66]. This releases β-catenin for nuclear signaling and, furthermore, by limiting cell-cell adhesion, it promotes cellular motility [21,67,68]. Cellular motility is crucial for interneuron migration, dendrite extension and synapse formation, and consequently, these processes are reduced by genetic disruption of *MET*[69,70]. *HGF-MET* signaling, therefore, contributes to neuronal differentiation, to development of cerebral cortex and cerebellum and to axon growth [66,69-71]. *MET* was shown to be associated with autism spectrum disorder in four independent family cohorts (reviewed by [72]), while one of risk alleles negatively regulated gene transcription [73]. A two-fold reduction of *MET* expression was found in *post mortem* temporal cortex of patients with autism [73]. In addition, two autism patients with a deletion CNV that encompassed *MET* were reported by Marshall *et al.*[74]. Finally, *MET* transcription is regulated by *FOXP2*, a further autism risk-gene [72]. These data indicate that reduction in *MET* function, perhaps paralleled by a reduction in Wnt signaling, contributes to autism susceptibility.

### Classical cadherins: CDH8 (16q21), CDH10 (5p14.1), CDH13 (16q23.3) and CDH15 (16q24.3)

The cadherin family is composed of more than 80 members of which about one quarter are so-called “classical” cadherins [75]. Classical cadherins form a complex with β-catenin and play a role in cell-cell adhesion [76]. Loss of function mutations in classical cadherins lead to decreased cell adhesion, an increase in cell motility, β-catenin release and an increase in canonical Wnt signaling [13]. Pagnamenta *et al.*[77] described two families with a rare 1.6 Mb microdeletion of the classical cadherin, CDH8, in which affected family members suffered from autism and learning disability. Also, the classical cadherin, CDH13, was found disrupted by a microdeletion, albeit thus far in a single patient only [52]. Furthermore, a genome-wide association study in 780 families with autism spectrum disorder produced a strong association signal for SNPs located between CDH9 and CDH10 on chromosome 5p14.1 [78]. Both cadherins are expressed in the brain, but the functional consequence of the SNPs was not investigated [78]. A further classical cadherin that may be implicated in autism is CDH15 [79]. The authors described patients with a microdeletion of 16q24.3, an area just distal to CDH15. The predicted functional consequence of haplotype-insufficiencies of these cadherins would be enhanced β-catenin release and activation of the Wnt pathway.

### TSC1 (9q34) and TSC2 (16p13.3)

Although mainly known for their role in the tuberous sclerosis syndrome, the tumor suppressors, tuberin (TSC2) and hamartin (TSC1), also participate in Wnt signaling. Both TSC1 and TSC2 were found to co-immunoprecipitate with axin and β-catenin [80]. Overexpression of TSC1 or TSC2 led to reduction of Wnt-induced β-catenin signaling, whereas mutations in TSC1 or TSC2, as found in tuberous sclerosis patients, led to increased canonical Wnt signaling [80]. In the brain, TSC1 and TSC2 have been implicated in cell body size, dendritic arborization, axonal outgrowth, neuronal migration, cortical lamination and spine formation [81]. The co-occurrence of autism and tuberous sclerosis has been recognized for decades and features of autism are present in up to half of the patients with tuberous sclerosis [81,82]. These findings support the contention that increased Wnt signaling may contribute to autism.

### *DISC1* (1q42)

The ‘disrupted in schizophrenia 1’ (*DISC1*) gene is disrupted by a balanced chromosomal translocation (1; 11) (q42; q14.3) in a Scottish family with a high incidence of bipolar disorder, major depression and schizophrenia [83]. *DISC1* can be considered an endogenous GSK3β inhibitor and in line with that activity, it promotes canonical Wnt to β-catenin signaling [27]. Expression in mice of the truncated *DISC1*-form identified in the Scottish family led to an attenuated neurite outgrowth of primary cortical neurons and behavioral hyperactivity [84]. To date, several linkage and association studies have confirmed the role of *DISC1* in neuropsychiatric disorders [85,86], including one study on autism and Asperger syndrome [87]. This study found an association between autism and a *DISC1* intragenic microsatellite marker and, furthermore, an intragenic three-SNP haplotype and Asperger syndrome [87]. About 3% of patients tested by Kilpinen and colleagues [87] had a double diagnosis of autism plus either schizophrenia or bipolar disorder. In fact, the same haplotype was found to be associated with schizophrenia and bipolar disorder [88]. Analysis of the promoter region of *DISC1* showed that *FOXP2* suppresses *DISC1* gene expression and protein levels [89]. Interestingly, autism-related mutations in FOXP2 diminished the suppressive effect on *DISC1* transcription [89]. So it could be that both diminished and enhanced *DISC1* function could contribute to autism spectrum disorder.

### APC (5q21-q22)

Adenomatous polyposis coli (APC) is a negative regulator of the canonical Wnt pathway and functionally disruptive mutations are known to predispose for colorectal cancer. Barber *et al.*[90] describe a patient who originally was referred for autism and who was subsequently found to carry an APC deletion and had developed rectal cancer. Zhou and colleagues [91] reported a two part study. In a retrospective study in 75 autism spectrum disorder patients and 476 controls an association was found between a SNP in the 3’ untranslated region of the *APC* gene and autism. In the second part, the authors performed a prospective study in a new set of 75 ASD patients and 280 new controls on 4 SNPs spanning the entire 100 kB gene. While the individual SNPs were not significantly associated, one of the possible haplotypes (TGAG) was [91]. Unfortunately, the functional consequence of the TGAG haplotype regarding Wnt pathway activity remains unknown.

### *DOCK4* (7q31.1)

Also, the *DOCK4* gene is located under the broad linkage peak on 7q31. Single nucleotide polymorphisms within *DOCK4* were associated with autism risk in different populations [41]. Further evidence for a role of *DOCK4* in autism was provided by the finding of a microdeletion CNV in an autistic sib-pair [41] and a deletion in a family with dyslexia [92]. *DOCK4* is a component of the β-catenin destruction complex and decreasing its expression by siRNA reduced Wnt-induced TCF transcriptional activity [93]. *DOCK4* is also involved in Wnt-induced activation of the GTPase, Rac, which is required for cell migration and synaptic function [93]. A study in rats showed that *DOCK4* is highly expressed in the hippocampus and *DOCK4* expression levels increase during periods of dendrite growth [94]. The data thus suggest that diminished *DOCK4* level (and presumably, function), as found in autism, suppresses Wnt signaling and dendrite growth.

### *AHI1* (6q23.3)

Joubert syndrome is characterized by ataxia, abnormal breathing patterns, sleep apnea, abnormal eye and tongue movements and hypotonia, as well as distinct malformations of cerebellum and brain stem. A high percentage of patients with Joubert syndrome have been diagnosed with autism spectrum disorder [95]. One of the first genes identified to be involved in the pathogenesis of this syndrome was ‘Abelson’s Helper Integration 1’ (*AHI1*) [96]. The *AHI1* gene was found to bear several mutations that give rise to non-functional variants of the encoded protein, Jouberin [96]. Dysfunction of Jouberin may thus lead to autism. In mice, *AHI1* was distributed throughout cytoplasm, dendrites and axons of neurons and was expressed from embryonal Day 10.5 onwards [97]. In particular, *AHI1* mRNA was expressed in cell bodies of midline-crossing neurons, providing an explanation for axonal abnormalities found in Joubert-syndrome [98]. Jouberin participates in the Wnt pathway by facilitating the nuclear accumulation of β-catenin [99], but it is currently not known if this is causally involved in the autism symptoms of Joubert syndrome patients.

### *EN2* (7q36)

*EN2* (engrailed-2) is involved in regionalization, patterning and neuronal differentiation of the mid- and hindbrain and is strongly expressed in these areas during embryonic development. A low level of expression is maintained in adulthood; for instance, in the hippocampus and cerebral cortex [100]. The transcription of *EN2* is enhanced by stimulation of the canonical Wnt pathway [101]. Several studies have shown an association between autism and SNPs in *EN2* (for review, see [102]). Two intronic SNPs were over-transmitted to affected individuals both singly and as haplotype in separate data sets from North American origin [102]. The risk haplotype (A-C) led to higher transcription of *EN2* than the opposite haplotype [102]. Remarkably, a study in Han Chinese autism cases confirmed *EN2* as a susceptibility gene, but found the A-C haplotype to be protective [103]. It is conceivable that both deficits and overexpression of *EN2* are disruptive for normal brain development. Knock-out mice, which lack both copies of *EN2*, display subtle cerebellar neuropathology and a behavior that could be interpreted as autism-like, for example, decreased play, reduced social sniffing and grooming, and reduced aggression [104]. For a better interpretation, one should also study *EN2*-overexpressing mice, but this has, to my knowledge, not been done. *EN1* and, to a lesser degree, *EN2* are expressed in dopamine neurons in the substantia nigra and ventral tegmental area [105].

### *CREBBP* (16p13.3)

CREB binding protein (CREBBP; 16p13.3) and its close analogue EP300 (22q13.2) are transcriptional co-activators of β-catenin [24]. Mutations and deletions of the *EP300* or *CREBBP* genes give rise to the Rubinstein-Taybi syndrome (characterized by broad thumbs and toes, short stature, distinctive facial features, impairments in cognitive and motor skills and micro- or macrocephaly; for review see [106]). Patients with deletions of CREBBP show cognitive impairment, autistic features and seizures [107]. Patients with a duplication of the 16p13.3 region, invariably encompassing the *CREBBP* gene, have also been described [108,109]. The behavioral phenotype of these patients is relatively mild, but can include autism spectrum disorder [109], speech deficits and moderate mental retardation [108]. Experiments in rats have shown that *CREB* activation is required for hepatocyte growth factor-induced dendritic arborization during brain development [71].

## Discussion (Part 2)

The literature reviewed above supports the contention that modification of genes affecting the activity of the canonical Wnt pathway can contribute to individual forms of autism spectrum disorder. Functionally active polymorphisms (SNPs) and copy number variations (CNVs) suggest both increases in Wnt signaling (SNPs in *MARK1* and *EN2* that increase gene transcription, *PTPRZ1* deletion, cadherin deletion-CNVs, *CREB* duplication-CNV), as well as decreases in Wnt signaling (SNPs and CNVs in MET that reduce transcription, deletion-CNVs in *DOCK4* and *CREB*, as well as disruptive SNPs in *DISC1* and *AHI1*). While reviewing the functions of the Wnt-pathway genes, the reader may notice a recurrent theme: their effect on cellular motility (neurite growth, spine and synapse formation). Thus, hepatocyte growth factor activation of MET, disruption of cadherin /β-catenin complexes and downstream activation of *CREB* are involved in neurite extension and development of the dendritic arbor. *TSC1* and *TSC2* play a role in these processes, too. *DOCK4* is expressed during periods of dendrite growth, while *AHI1* is important for midline-crossing axons. A crucial observation is that both overexpression and genetic silencing of *MARK1* resulted in too short dendrites. This indicates that both too much and too little Wnt pathway activity is deleterious for dendrite growth. Consequently, both hyperactivation and hypoactivity of the Wnt pathway will negatively affect cognitive function and, since language development is a cognitive skill, it is conceivable that linguistic capabilities are reduced too.

## Medications that influence the canonical Wnt pathway

A study by Rasalam *et al.*[110] noted that 1 out of 10 children born from mothers taking antiepileptic medication had social, behavioral and language difficulties. Valproate was the drug that was most commonly associated with autistic disorder [110,111]. This is supported by data from animal studies. When rats were prenatally (Day 12.5) exposed to a single dose of sodium-valproate, after birth they exhibited a lower sensitivity to pain, but a higher sensitivity to non-painful stimuli [112]. Furthermore, the animals displayed hyper-locomotion and stereotypy and lower exploratory activity, a decreased number of social behaviors and longer latency to social behavior. All behaviors appeared prior to puberty. Prenatal valproate use in rats is considered an animal model of autism [112]. Interestingly, prenatal exposure to valproate led to increases in mRNA and protein levels of WNT1 and WNT2 in the prefrontal cortex and hippocampus, and genes under transcriptional control of the Wnt pathway (for example, *EN1*, *cyclin D1*) were up-regulated [113]. Other medications which are suspected of contributing to autism prevalence are antidepressants, in particular the serotonin re-uptake inhibitors (SSRIs) [114]. A prospective population-based study by Croen *et al.*[115], in which prenatal SSRI exposure of autistic children was compared to SSRI exposure in control children, found a doubling of the risk of autism when the mother took an SSRI during the year before delivery, while the most pronounced risk was seen when exposure occurred during the first trimester. The authors concluded that exposure to SSRIs during the first trimester of pregnancy modestly increases the risk for autism spectrum disorder. Rats that were exposed to the SSRI, citalopram, during postnatal Days 8 to 21 displayed altered branching characteristic in hippocampal and neocortical neurons, had reduced myelination of callosal axons and, furthermore, showed impaired social behavior and response to novelty [116]. This result shows that alterations in central serotonin levels may interfere with normal brain development. Subchronic treatment of rats with the antidepressants citalopram, fluoxetine, venlafaxine and atamoxetine increased the expression of several Wnt-pathway genes; the effect shared by all antidepressants was an increase in *WNT2-* expression, involving both mRNA and protein levels [117]. It is evident that antidepressant-induced Wnt signaling has the propensity to influence brain development, and reviewed data provide further support for the contention that altered Wnt pathway activity is a risk factor for autism spectrum disorder.

## Discussion (Part 3)

When particular medications like anticonvulsants or antidepressants can increase risk for autism, it is conceivable that appropriate medications can reduce autism risk. The information reviewed above suggests that alterations in the activity of the canonical Wnt pathway could contribute to autism risk and, consequently, pharmacotherapeutic correction of the aberrant pathway activity might help to improve symptoms. It seems that both hyperactivity and hypoactivity can generate symptoms, implying that patients have to be stratified according to their Wnt pathway activity status before pharmacotherapy can be initiated. How should this stratification be done? One possibility would be to stratify according to skull size. Unfortunately, the neuro-developmental mechanisms that regulate brain and skull growth are multiple, and involve not only the canonical Wnt pathway, but also growth-factor pathways like the ERK-mitogen-activated protein-kinase pathway, the PI3K-PKB-mTOR pathway, the Sonic hedgehog pathway (and so on) and, also, include nuclear receptor activators like retinoic acid, thyroid hormone, corticosteroids and gonadal steroids (for review see [118]). A more direct way to determine canonical Wnt-pathway activity would be to screen for proteins regulated by Wnt (for example, c-Myc, cyclin-D1 or ADAM10) [119-123]. Blood plasma levels of such proteins might become a biomarker for pathway activity.

## Consequences for the treatment of autism spectrum disorders

Given the importance of the canonical Wnt pathway for the development of the brain and other organs, modifying its activity, in particular in young children, is a quite hazardous enterprise. Furthermore, since autism is a neurodevelopmental disorder, it cannot be excluded that drug treatment will be only effective during a narrow period, while treatment outside this critical period is inactive [124], and thus dangerous. Therefore, pharmaco-therapeutic treatment would only be justifiable if a valid surrogate marker for canonical Wnt pathway activity would be available. Under such circumstances, one could then consider treatment with ‘mild’ pathway-modifying drugs. Lithium is such a drug: it activates the canonical Wnt pathway [125] without at the same time raising cancer risk [126,127]. Patients with Williams-Beuren syndrome, *MET* mutations, *DOCK4* microdeletions or Joubert syndrome might indeed benefit from lithium treatment; however, it would probably be contra-indicated in patients with a cadherin haplo-insufficiency, tuberous sclerosis or MARK1 mutations. ‘Soft’ treatments of autism disorder related to Wnt pathway-hyperactivity can in principle be found among anti-cancer drugs. The non-steroidal, anti-inflammatory compound sulindac could be an option. This compound inhibits polymerization of dishevelled [128] and consequently inhibits β-catenin signaling [128-130]. Interestingly, the activity of sulindac is not related to COX-inhibition since sulindac-sulphone, a metabolite devoid of COX-inhibition, is equally effective as a Dvl-inhibitor as sulindac itself [128]. However, at present it is clearly premature to propose sulindac as treatment for autism (respectively, autism spectrum disorders).

## Conclusion

Taken as a whole it seems safe to conclude that the activity of the canonical Wnt pathway is altered at least in a subset of patients with autism spectrum disorder. Whether correction of the deviant pathway activity leads to symptomatic improvement remains unknown. It is important to realize deviations from the optimum in both directions seem to increase the risk for autism spectrum disorder. This implies that patients, depending on their Wnt pathway activity, will have to be treated differentially.

## Abbreviations

ADAM: A desintegrin and metalloproteinase; ADHD: Attention deficit hyperactivity disorder; AHI: Abelson’s helper integration; APC: Adenomatous polyposis coli; BCL9: B-cell lymphoma-9); CBP: CREB binding protein; CDH: Cadherin; CK: Casein-kinase; CNV: Copy number variation; DISC1: Disrupted in schizophrenia-1; DOCK4: Dedicator of cytokinesis-4; EN: Engrailed; FOXP: Forkhead box P; FZD: Frizzled; GSK3: Glycogen-synthase kinase-3; HEK293: Human embryonic kidney 293; HGF: Hepatocyte growth factor; LRP: Low-density lipoprotein receptor-related protein; MARK: Microtubule affinity regulating kinase; PP: Protein phosphatase; PTPR: Protein tyrosine phosphatase receptor-type; SNP: Single nucleotide polymorphism; SSRI: Selective serotonin re-uptake inhibitor; TCF: T-cell factor; TDT: Transmission disequilibrium test; TSC: Tuberosclerosis; WBS: Williams-Beuren syndrome; Wnt: Wingless-type mouse mammary tumor virus integration site; WIF: Wnt inhibitory factor.

## Competing interests

At the time of writing, the author’s salary was paid by Novartis. The author owns Novartis stock.

## Author’s contribution

HOK searched, read and summarized the literature, wrote the article and created the figure.

## Author’s information

HOK worked for 29 years in the nervous system department of Novartis and led several research programs (including GSK3-inhibitors and AMPA-receptor antagonists). He is now retired.
